# YL143, a novel mutant selective irreversible EGFR inhibitor, overcomes EGFR^L858R, T790M^‐mutant resistance in vitro and in vivo

**DOI:** 10.1002/cam4.1392

**Published:** 2018-03-13

**Authors:** Zhang Zhang, Jian Zou, Lei Yu, Jinfeng Luo, Yan Li, Zhengchao Tu, Xiaomei Ren, Hongcheng Wei, Liyan Song, Xiaoyun Lu, Ke Ding

**Affiliations:** ^1^ School of Pharmacy Jinan University 601 Huangpu Avenue West Guangzhou 510632 China; ^2^ University of Chinese Academy of Sciences 19 Yuquan Road Beijing 100049 China; ^3^ Guangzhou Institutes of Biomedicine and Health Chinese Academy of Sciences 190 Kaiyuan Avenue, Guangzhou Science Park Guangzhou 510530 China; ^4^ State Key Laboratory of Drug Research Shanghai Institute of Materia Medica Chinese Academy of Sciences Shanghai 201203 China; ^5^ The First Affiliated Hospital Jinan University 613 Huangpu Avenue West Guangzhou 510630 China

**Keywords:** EGFR inhibitor, lung cancer, pharmacokinetic, selectively, T790M mutation

## Abstract

YL143 was identified as a novel wild‐type sparing EGFR^T790M^ inhibitor with good pharmacokinetic properties. It potently suppresses EGFR^L858R/T790M^ with an 50% inhibitory concentration (IC_50_) value of 2.0 ± 0.3 nmol/L, but is approximately 92‐folds less potent against EGFR^WT^ kinase. YL143 suppresses cellular proliferation and induces G0/G1 phase arrest and apoptosis in H1975 cells with EGFR^L858R/T790M^ mutation at 30 nmol/L. It also exhibits acceptable pharmacokinetics (PK) parameters with an oral bioavailability value of 25.0% after oral administration in rats and exhibits promising antitumor efficacy in a gefitinib‐resistant human H1975 xenografted model after oral administration of 30 mg/kg/day. These data supported that YL143 could be a promising lead compound for overcoming clinical EGFR^T790M^ resistance of patients with non‐small‐cell lung cancer (NSCLC).

## Introduction

Lung cancer is a leading cause of cancer‐related death worldwide 1, 2. Non‐small‐cell lung cancers (NSCLC) that comprise almost 85% of all human lung tumors include squamous cell carcinoma, adenocarcinoma, and large‐cell lung cancer 3, 4. Genetic aberrations within the tyrosine kinase domain of the epidermal growth factor receptor (EGFR) such as L858R mutation and E746‐A750 deletion have been observed in approximately 30% patients with NSCLC and have been identified as key drivers of NSCLC progression 5, 6. Abnormal activation of EGFR leads to overactivation of downstream Ras‐Raf‐MAPK and PI_3_K‐AKT pathways associated with tumor growth and poor outcome 4, 7.

The first‐generation EGFR kinase inhibitors, for example, gefitinib and erlotinib, produce reliable responses and survival benefits in patients with NSCLC with EGFR activating mutation. However, the rapid emergence of acquired resistance limits their clinical efficacy 8. Approximately 50% of resistant patients harbor a secondary threonine^790^ to methionine^790^ mutation in EGFR kinase domain 9. The second‐generation inhibitors such as afatinib were developed consequently with the aim to overcome the EGFR^T790M^ mutation‐mediated resistance 10. However, most of the second‐generation inhibitors display relatively low therapeutic window in resistance patients because of their poor selectivity over wild‐type (WT) EGFR and/or poor pharmacokinetics parameters. In order to solve the “on‐target” toxicity, several third‐generation EGFR^T790M^‐mutant selective inhibitors, for example, WZ4002 11, CO1686 (Rociletinib) 12, AZD9291 (Osimertinib) 13, 14, and HM61713 (BI1482694, Olmutinib) 15, were developed. Significantly, osimertinib had been approved by the FDA to treat the patients who exhibit resistance to gefitinib and erlotinib therapy and harbor EGFR^T790M^ mutation. The latest clinical data demonstrated that osimertinib improved PFS (progression‐free survival) from 10.2 to 18.9 months 16. However, the clinical investigation of CO1686 was terminated because of the strong IGF1R inhibitory activities of its metabolites causing hyperglycemia in about 40% patients 17, 18, 19.

Considerable efforts have been devoted to the development of third‐generation EGFR inhibitors, and several series of small molecules with distinct chemical scaffolds were designed and synthesized as novel wild‐type sparing EGFR inhibitors from our group 20, 21, 22, 23. We have reported XTF262 as a highly potent and specific EGFR^T790M^ inhibitor with an 50% inhibitory concentration (IC_50_) value of 0.8 nmol/L against EGFR^L858R/T790M^ 20. However, this compound possessed a poor oral bioavailability value below 10% and exhibited little antitumor efficacy in a H1975 xenograft mouse model. Further structure‐guided optimization helped us to obtain a new promising derivative YL143 which not only displayed strong and specific inhibition against EGFR^T790M^ but also exhibited obviously improved pharmacokinetics (PK) properties with a rat bioavailability of 25.0% and good antitumor activity in vivo xenograft model.

## Materials and Methods

### Reagents and antibodies

YL143, *N*‐(3‐(2‐((2‐Methoxy‐4‐(4‐methylpiperazin‐1‐yl)phenyl)amino)‐5‐methyl‐7‐oxopyrido [2,3‐*d*]pyrimidin‐8(7*H*)‐yl)‐5‐methylphenyl)acrylamide, was designed and synthesized in our laboratory. The compound was dissolved in Dimethyl sulfoxide (DMSO) (Sigma‐Aldrich) at a concentration of 10 mmol/L and stored at −20°C. Primary antibodies against EGFR (#4267), phosphor‐EGFR (#2234), ERK1/2 (#4695), phosphor‐ERK1/2 (#4370), CDK2 (#2546), CDK4 (#2906), cyclin D2 (#3741), cyclin E (#4129), caspase‐3 (#9665), caspase‐9 (#9502), Glyceraldehyde‐3‐phosphate dehydrogenase (GAPDH) (#2118), and horseradish peroxidase (HRP)‐linked secondary antibodies against rabbit or mouse were purchased from Cell Signaling Technology (Boston, MA).

### Cell culture

H1975, HCC827, A549, and A431 were purchased from American type culture collection (ATCC). All cell lines were grown in RPMI‐1640 or DMEM supplemented with 10% FBS and 1x penicillin/streptomycin solution (Invitrogen) in a humidified CO_2_ incubator at 37°C. All cells were in the logarithmic growth phase at the initiation of experiments.

### In vitro kinase activity assay

All EGFR (WT, L858R, L858R/T790M, etc.) recombinant human protein was purchased from Invitrogen. The kinase activity assay was performed and optimized according to the instructions of Z’‐LYTE^™^ kinase assay kits from the manufacturer (PV4879, Life, America). In our experiments, 10 concentration gradients from 0.01 nmol/L to 1.0 *μ*mol/L were used for all the compounds.

### Molecular docking studies

All the procedure was performed in Maestro 9.9 (Schrodinger LLC). The crystal structure of EGFR was taken from protein database bank (PDB ID 5GMP). The covalent bond between the inhibitor and the protein was deleted. Then, the protein was processed using the “Protein Preparation Wizard” workflow in Maestro 9.9 (Schrodinger LLC) to adding bond orders and hydrogens. All het atm residues and crystal water molecules beyond 5 Å from het group were removed. YL143 was built by in LigPrep module using OPLS‐2005 force field. Glide module (covalent docking) was used as docking program. Michael addition was chosen as the reaction type. Cys797 was chosen as the reactive residue. The docking box was placed on the centroid of the binding ligand in the optimized crystal structure as described above. Covalent docking approach of Glide was adopted to dock YL143 to EGFR with the default parameters.

### In vitro cell growth assay

Cells were seeded into 96‐well plates at a density of 1500~3000/well in complete medium overnight. Then, cells were treated with various concentrations (0.01~1000 nmol/L) of YL143 for additional 3 days. Viable cell numbers were determined by Cell Counting Kit 8 (CCK8, CK04, Dojindo Laboratories, Japan). Each assay consisted of three replicate wells and repeated at least three times. IC_50_ values were calculated by inhibitor–response nonlinear regression curve fitting using GraphPad Prism 5.0 software (developed by Dr Harvey Motulsky, San Diego, CA 92037 USA). Each IC_50_ value was expressed as mean ± SD.

### H1975 tumor xenograft

Male CB17‐SCID mice were purchased from Vital River Laboratory Animal Technology Inc. (Beijing, China). H1975 cells (5 × 10^6^/mouse) were injected subcutaneously in the right flanks of mice. When the mean tumor volume reached 100–200 mm^3^, tumor‐bearing mice were randomized and treated with YL143 (30 mg/kg), CO1686 (30 mg/kg), and vehicle (10 mL/Kg) for the 18 consecutive days once daily by oral gavage, respectively. All the compounds were suspended in 0.5% CMC‐Na aqueous solution. The tumor volume and body weight of animals were measured once every 2 days. Tumor volume was calculated as the *L* × *W* ^2^/2 (*L* and *W* are the length and width of the tumor, respectively). All animal experiments were approved by the Institutional Animal Use and Care Committee of Guangzhou Institute of Biomedicine and Health, Chinese Academy of Science.

### Western blot analysis

Cells were treated with various concentrations of compound for designed time. Then, cells were lysed using 1 × SDS sample lysis buffer (CST recommended) with protease and phosphatase inhibitors. Cell lysates were loaded and electrophoresed onto 8–12% SDS‐PAGE gel, and then, separated proteins were transferred to a PVDF film. The film was blocked with 5% fat‐free milk in TBS solution containing 0.5% Tween‐20 for 4 h at room temperature and then incubated with corresponding primary antibody (1:1000–1:200) overnight at 4°C. After washing with TBST, HRP‐conjugated secondary antibody was incubated for 2 h. And the protein signals were visualized by ECL Western Blotting Detection Kit (Thermo Scientific, Waltham, MA) and detected with Amersham Imager 600 system (GE, America).

### Pharmacokinetic study

Sprague Dawley (SD) rats (180–220 g) were fasted overnight before drug administration. They could eat food and drink water freely during the study. Rats were grouped and treated with 25 and 5 mg/kg of YL143 in vehicle *via* oral gavage and i.v., respectively. Eyeground vein blood samples (0.20 mL) were collected from each animal at designed time point postdose (5, 30 min, 1, 2, 4, 8, 12, 24, 36, 48, 60, and 72 h.). Blood samples were centrifuged within 10 min at 900 g, and then, plasma was separated and stored at 4°C until analysis. Drug concentration was quantitatively detected using an API 300 mass spectrometer (Applied Biosystems, Canada) with TurboIonSpray source interface. Data acquisition and quantitation were performed using Analyst 1.4 (Applied Biosystems, Canada). All pharmacokinetic parameters were calculated by DAS 2.0 using a noncompartment model (Clinical drug research center of Shanghai University of Traditional Chinese Medicine, Shanghai, China).

### Transferase‐mediated deoxyuridine triphosphate‐biotin nick end labeling (TUNEL) staining

Resected mouse tumors were fixed in 4% paraformaldehyde solution (Jingxin Biotech, China), then paraffin embedded, and sectioned for TUNEL staining analysis according to the instructions of the manufacturer (11684817910, Roche, Germany).

### Statistical analysis

All data are displayed as the mean ± SD of at least three experiments. Differences between groups were involved one‐way ANOVA with post hoc intergroup comparison using Tukey test using SPSS 17.0 (SPSS, Inc., Chicago, IL, USA). Differences with *P* < 0.05 were considered significant and marked as*.

## Results

### YL143 is a novel mutant selective, irreversible inhibitor for EGFR

**Figure 1:**
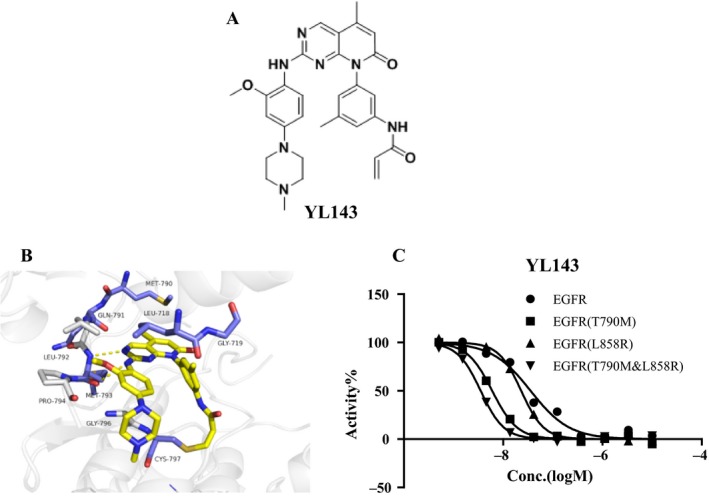
YL143 is a novel irreversible EGFR inhibitor. (A) Structure of YL143 and XTF262. (B) The binding mode of YL143 with EGFR predicted by the molecular docking simulations. (C) Kinase inhibitory activity of YL143 on EGFRWT, EGFRL858R, EGFRT790M and EGFRL858R/T790M by FRET‐based Z′‐Lyte assay.

YL143 (Fig. 1A) possesses a novel chemical structure optimized from XTF262 (Fig. S1), which retains its activity and selectivity on EGFR^T790M^ and improves the oral bioavailability (Table 3, S2).

The kinase inhibitory activities of YL143 against EGFR^WT^, EGFR^L858R^, EGFR ^Del 19^, EGFR^L858R/T790M^, and EGFR ^Del 19, T790M^ were evaluated using a well‐established FRET‐based Z’‐Lyte assay. Three reported EGFR inhibitors, that is gefitinib, CO1686, and osimertinib, were used as the positive controls. Under the experimental conditions, all of the drugs exhibited similar activities and selectivity profiles to the reported data 12, 13 (Table 1, Table S1). YL143 potently inhibited EGFR^L858R/T790M^ with an IC_50_ value of 2.0 ± 0.3 nmol/L, which is more potent than the reported third‐generation EGFR inhibitors (i.e., CO1686 and osimertinib) by a factor of 5–7 in a parallel comparison (Fig. 1C, Table 1). Furthermore, YL143 displayed a 92.6‐fold selectivity between the L858R/T790M mutant and the wild‐type EGFR, while the selectivity of CO1686 and osimertinib was 10.7‐ and 18.3‐fold, respectively.

**Table 1:** In vitro EGFR tyrosine kinases activities of YL143

nmol/L (AV ± SD)	Gefitinib	CO1686	AZD9291	YL143
EGFR^WT^	10.7 ± 0.4	157.0 ± 22.4	173.7 ± 29.3	185.3 ± 43.4
EGFR^L858R^	1.3 ± 0.1	23.7 ± 2.1	6.4 ± 1.6	49.1 ± 4.2
EGFR^Del 19^	3.8 ± 1.5	90.7 ± 30.0	3.4 ± 1.7	81.5 ± 10.3
EGFR^T790M^	134.6 ± 17.3	17.2 ± 1.4	7.9 ± 2.3	7.8 ± 1.1
EGFR^L858,T790M^	118.2 ± 7.1	14.6 ± 5.5	9.5 ± 1.8	2.0 ± 0.3
IGF1R	>1000	47.5 ± 8.4	276.8	>1000
IR	3.5 ± 0.5	12.8 ± 0.8	235	7.5 ± 0.1

EGFR activity assays were performed using the FRET‐based Z′‐Lyte assay according to the manufacturer's instructions. The compounds were incubated with the kinase reaction mixture for 1.5 h before measurement. The data are means from two independent experiments, and the variations are below 20%.

**Figure 2:**
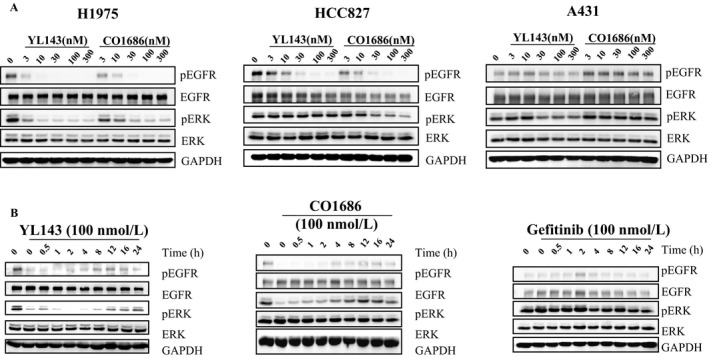
YL143 suppresses EGFR signaling pathway in H1975 cells (A) YL143 potently inhibits the activation of EGFR signals in H1975 NSCLC. Cells were treated with or without compound YL143 for 4 h at indicated concentration, respectively. Cells were harvested for Western blot analysis. (B) Wash‐out assay demonstrates the irreversible binding of YL143 with EGFR L858R/T790M. NCI‐H1975 cells were treated with or without compound YL143(100 nmol/L) for 4 h, and then the medium with compound was removed and fresh medium was added. At the indicated time points, cells were harvest and proteins were extracted and subjected to Western blot analysis.

XTF262 has been proved to be an irreversible EGFR inhibitor through inhibitor washout assay and in vitro mobility shift assay 21. To prove the irreversible binding mode of YL143 with EGFR^L858R/T790M^, a compound washout assay was performed on H1975 cells in vitro. The Western blotting (WB) results displayed that the activation of EGFR^L858R/T790M^ and ERK was constantly inhibited and no EGFR^L858R/T790M^ phosphorylation recovery was observed for 24 h after YL143 was withdrawn, strongly implying that the YL143 irreversibly bound to the EGFR^L858R/T790M^ (Fig. 2B).

### Molecular docking simulation

The computational docking studies suggested that YL143 bound to the ATP binding site of EGFR with a “U‐shaped” conformation (Fig. 1B). The pyrido[2,3‐*d*]pyrimidine‐7‐one core formed bidentate hydrogen bonding interactions with the “hinge” residue Met793, and the acrylamide covalently bound to the Cys797. The 5‐methyl at the pyrido[2,3‐*d*]pyrimidine‐7‐one was slipped into the “gatekeeper” residue Met790 which likely contributed to the selectivity of EGFR^T790M^ over the wild‐type EGFR. In addition, the right‐arm 3‐menthyl phenyl ring YL143 was pointed to hydrophobic core formed by residues Leu718 and Gly719. The aniline ring made hydrophobic interaction with Gly796 and Pro794, and the methoxy group extended to pocket formed by Leu 792 and Pro 794 in the hinge region.

### YL143 selectively inhibited proliferation of EGFR‐mutant cell lines

The proliferation inhibitory activity of YL143 was investigated in H1975 (EGFR^L858R/T790M^), HCC827 (EGFR^del 19^), A549 (EGFR^WT^), and A431 (EGFR^WT^) cells. It was shown that YL143 strongly suppressed the proliferation of H1975 and HCC827 cells with IC_50_ values of 45.2 ± 27.3 and 21.1 ± 9.9 nmol/L, respectively (Table 2). However, its activity against A431 and A549 cancer cells was minor with IC_50_ value of over 1.0 *μ*mol/L. These cellular data are highly consistent with the compounds’ potent and selective suppression against EGFR ^T790M^ kinase.

**Table 2:** Antiproliferative activities of YL143 and drugs

nmol/L (AV ± SD)	H1975	HCC827	A431	A549
Gefitinib	>1000	6.2 ± 4.1	>1000	>1000
AZD9291	40.3 ± 16.6	3.9 ± 3.5	>1000	>1000
CO1686	41.8 ± 25.1	67.2 ± 19.5	>1000	>1000
YL143	45.2 ± 27.3	21.1 ± 9.9	>1000	>1000

The antiproliferative activities of the compounds were evaluated using CCK‐8 assay. The cells were treated with compound or 0.1% DMSO for 72 h. The data were means from at least three independent experiments.

### YL143 inhibited EGFR signaling pathways in H1975 cell lines

The selective EGFR ^L858R/T790M^ inhibition of YL143 was investigated in H1975, HCC827, A549, and A431 cancer cells harboring different statuses of EGFR by Western blotting analysis (Fig. 2A). It was displayed that YL143 dose‐dependently inhibited the phosphorylation of EGFR and the downstream proteins Erk without obviously affecting their total proteins’ expression level in H1975 cells; however, its effect on EGFR signal pathway in A431 cells was obviously less potent.

### YL143 induced G0/G1 phase arrest and apoptosis in H1975 cell lines

**Figure 3:**
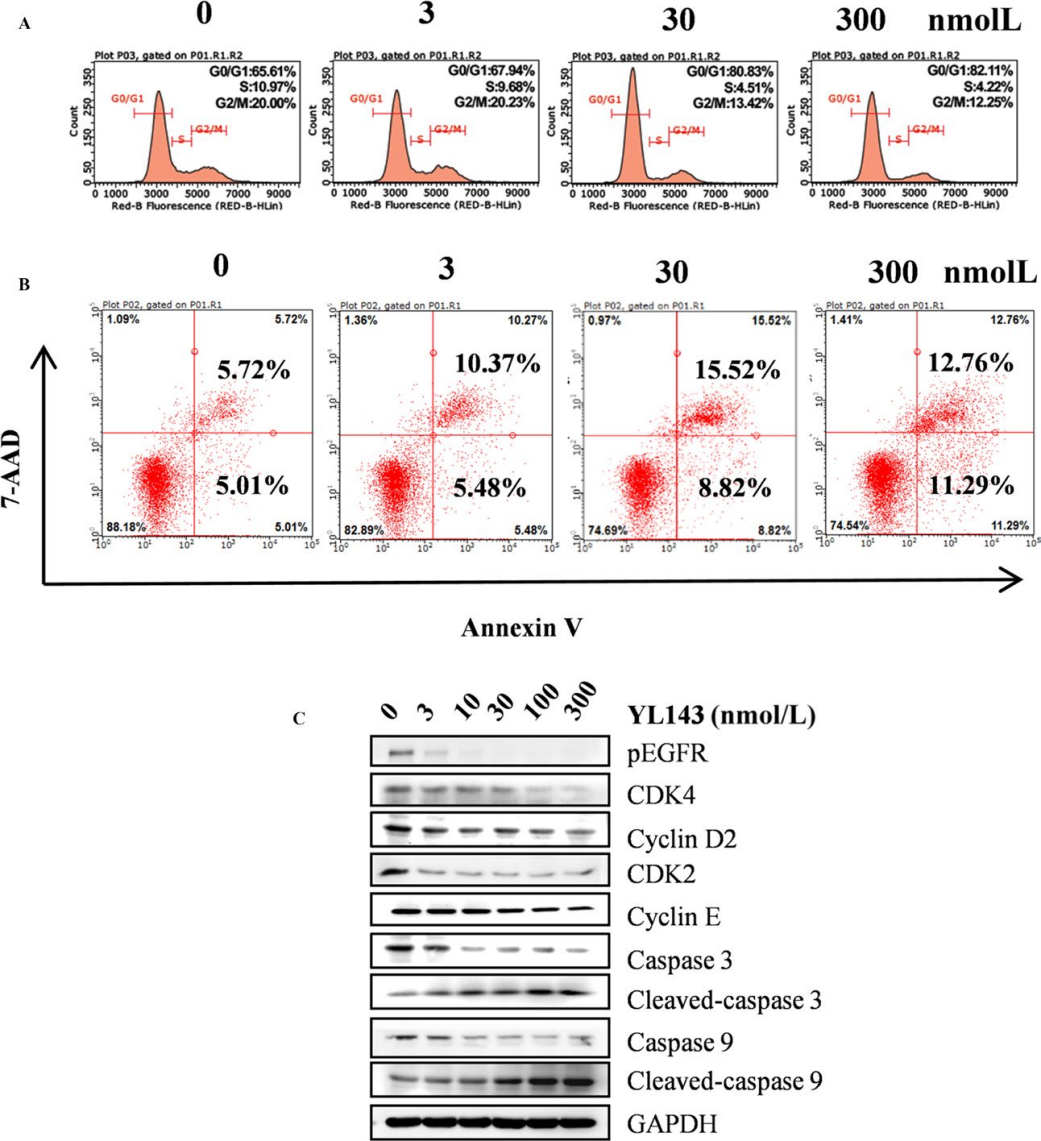
YL143 induces G0/G1 cell cycle arrest and apoptosis in H1975 cells. (A) YL143 induces G0/G1 cell cycle arrest in H1975 cells H1975 cells were treated with 3, 30 or 300 nmol/L for 48 h before DNA labeling by propidium iodide and cell cycle analysis by flow cytometry. (B) YL143 induces apoptosis in H1975 cells. H1975 cells were treated with 3, 30 or 300 nmol/L for 48 h before DNA labeling by Annexin V and 7‐AAD and cell apoptosis analysis by flow cytometry. (C) The effect of YL143 on the expression of CDK4, Cyclin D2, CDK2, Cyclin E, Caspase‐9 and Caspase‐3 was tested by western blotting. The cells were treated with YL143 from 3 to 300 nmol/L for 48 h. Representative results are shown and similar results were obtained in three other independent trials.

The effect of YL143 to induce cell cycle arrest and apoptosis in H1975 cells was investigated by flow cytometry analysis. It was shown that YL143 dose‐dependently induced G0/G1 phase arrest (Fig. 3A) and apoptosis (Fig. 3B) in H1975 cells. Treatment with 300 nmol/L of YL143 for 48 h led to 82.11% G0/G1 phase arrest and 34.1% apoptosis. Western blotting analysis also showed that YL143 dose‐dependently decreased the protein levels of CDK2, CDK4, cyclin D2, and cyclin E and the activating cleavage of caspase‐3 and caspase‐9 in H1975 cells. (Fig. 3C).

### Pharmacokinetics study

Further investigation revealed that YL143 significantly improved pharmacokinetic properties compared with the previous lead molecule XTF262. The PK properties of YL143 were evaluated in rats after single administration (5 mg/kg for intravenous injection, 25 mg/kg for oral). It was shown that YL143 exhibited an optimal plasma exposure (C_max,_ 160.32 *μ*g/L, 297 nmol/L) and a half‐life of 6.1 h after single oral dosing 25 mg/kg (Table 3). YL143 also displayed a good oral bioavailability of 25.0%. The C_max_(297 nmol/L) was about 4.3‐fold higher than IC_50_ (45.2 ± 27.3 nmol/L) in H1975 cells.

**Table 3:** Preliminary pharmacokinetic profiles of YL143 in rats

Parameter	Route (mg/Kg)	
	P.O.(25)	I.V.(5)
AUC_0‐∞_ (*μ*g/L*h)	2343.03	1877.85
T_1/2_ (h)	6.13	1.36
T_max_ (h)	6.67	0.08
C_max_ (*μ*g/L)	160.32	3267.84
BA (%)	24.95	/

T_1/2_, half‐life; C_max_, maximum concentration; T_max_, time of maximum concentration; AUC _0‐∞_, area under the plasma concentration–time curve; F, oral bioavailability.

### YL143 suppressed the H1975 xenograft tumor growth in vivo

**Figure 4:**
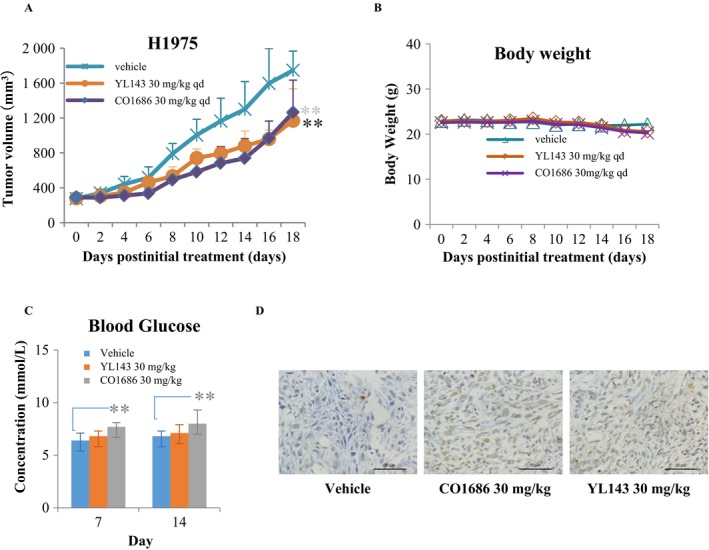
In vivo effect of YL143 on tumor volume and blood glucose in H1975 xenograft model. (A) Antitumor efficacy of YL143 in a human NSCLC (H1975) xenograft mouse model. (B) Effect of YL143 on body weight in a human NSCLC (H1975) xenograft mouse model. (C) Effect of YL143 on blood Glucose were detected by biochemical analysis at 7 and 14 day after dosing vehicle, CO1686 or YL143. (D)TUNEL staining of H1975 tumors harvested from the mice after dosing vehicle ,CO1686 or YL143 for 18 days. Mice were orally dosed once daily (qd) for 18 days with vehicle ,CO1686 or YL143 (30 mg/kg, po, qd) . Tumors and body weight were measured every other day (*P < 0.05, **P < 0.01).

We further evaluated the in vivo antitumor efficacy of YL143 on H1975 cancer cells using CB17‐SCID mouse xenograft models. The mice were administrated with vehicle, CO1686, or YL143 once daily *via* oral gavage (30 mg/kg/day, respectively) for 18 consecutive days. YL143 was well tolerated in all of the tested groups with no mortality or significant loss of body weight observed during experiment (Fig. 4B). It was shown that YL143 exhibited similar antitumor efficacy to CO1686 in the H1975 xenografted mice (Fig. 4A). TUNEL stain of the tumor tissue showed that YL143 induced apoptosis in H1975 xenograft model (Fig. 4D).

## Discussion

Epidermal growth factor receptor is a well‐validated target for lung cancer therapy. The reversible first‐generation ATP‐competitive EGFR inhibitors, that is, gefitinib and erlotinib, have achieved excellent clinical benefit in patients with NSCLC harboring EGFR mutations. The drugs significantly improve the median progression‐free survival (PFS) from 4.6 to 13.1 months in patients with NSCLC with EGFR exon 21 L858 mutation (EGFR^L858R^) or exon 19 deletion (EGFR^del19^) 24. However, approximately 60% patients suffered from drug resistance after a treatment period due to a secondary acquired EGFR^T790M^ mutation in exon 20 9, 25. The second‐generation EGFR inhibitors, such as afatinib 26, bind irreversibly with EGFR *via* a cysteine residue (C797) in the kinase domain. But afatinib demonstrated limited clinical efficacy in the context of acquired EGFR T790M mutation because of its relatively low maximum tolerated dose (MTD) caused by the nonselective strong suppression against wild‐type EGFR. Aim to resolve the ineffectiveness of second‐generation inhibitors, the third‐generation drugs that selectively inhibited EGFR^T790M^ mutants were developed. Several third‐generation EGFR inhibitors such as WZ4002 11, CO1686 12, and HM61713 15 have been developed into different stages of clinical investigation, and AZD9291 has been approved by US FDA for EGFR^T790M^ resistance patients. It has become a reliable strategy to overcome mutant resistance of NSCLCs by developing wild‐type sparing EGFR^T790M^ inhibitors.

We have previously reported XTF262 21 which displayed potent and specific EGFR^L858R/T790M^ inhibition with approximately 100‐fold selectivity over the wild‐type EGFR. However, this molecule possesses unacceptable PK properties and displayed poor antiproliferation efficacy in H1975 xenograft model. Chemical structure analysis suggested that the highly planar configuration contributes greatly to its low aqueous solubility. Aim to improve the PK profiles, further structural optimization was conducted based on a high‐resolution X‐ray structure of XTF262‐EGFR^T790M^ complex. The efforts led to the discovery of a new derivative YL143, as a highly potent and selective EGFR^T790M^ inhibitor with improved PK profiles. YL143 possessed a reasonable oral bioavailability value of 25.0% in rats and also retained a strong EGFR^L858R/T790M^ inhibitory activity with an IC_50_ value of 2.0 ± 0.3 nmol/L and superior target selectivity (approximately 92.6‐fold over the wild‐type kinase). In addition, the compound also exhibited promising antiproliferative activity against H1975 cells an IC_50_ value of 45.2 ± 27.3 nmol/L and demonstrated promising antitumor efficacy in a H1975 xenograft model without significantly toxic signs during treatment.

In the clinical study, hyperglycemia was found in 47% and six of 238 patients for CO1686 and osimertinib, respectively 4. The hyperglycemia required dose reduction in CO1686 or addition the oral hypoglycemic agent. Further analysis suggested the hyperglycemia is due to a CO1686 metabolite (M502) possessing insulin‐like growth factor receptor‐1 inhibitory activity 4. YL143 displayed comparable in vivo antitumor effect to that of CO1686 but weaker hyperglycemia side effect (Fig. 4C), suggesting that YL143 might possess a better therapeutic window. It has reported that IGF1 played its functions in glucose metabolism through binding IR and IGF1R heterodimeric receptors or its own receptor IGF1R 27, 28. YL143 displayed no IGF1R kinase inhibitory activity (IC_50_ value >1.0 *μ*mol/L) but exhibited an IC_50_ value of 7.5 ± 0.1 nmol/L against IR under our experimental condition (Table 1). Further structure modification of YL143 to decrease IR kinase activity is necessary for avoiding potential hyperglycemia adverse effects.

In conclusion, we have developed a novel EGFR inhibitor YL143, which displayed highly potent and specific EGFR^T790M^ inhibition, and improved the oral bioavailability. YL143 also exhibited antiproliferative effect in H1975 cells and animal xenografts. YL143 may serve as a new potential lead compound for drug development overcoming EGFR^L858R/T790M^ resistance for NSCLC patients’ treatment.

## Conflict of Interest

None declared.

## Supporting information


**Figure S1**. Structure of XTF262.

**Table S1**. Anti‐proliferative activities of YL143 and drugs in BaF3 stable cells.

**Table S2**. In vitro kinases activities of YL143.
