# Supplementary figures and images for: YL143, a novel mutant selective irreversible EGFR inhibitor, overcomes EGFRL858R, T790M‐mutant resistance in vitro and in vivo
Source: Cancer Med. 2018 Mar 13;7(4):1430–9. doi: 10.1002/cam4.1392 (PMC5911580; doi:10.1002/cam4.1392)

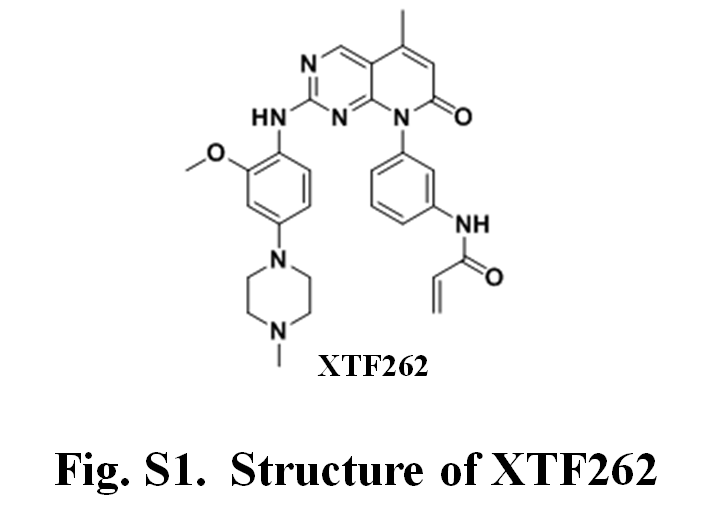

Supplement: Supplementary file 1 — Figure S1. Structure of XTF262. [file CAM4-7-1430-s001.tif]
