# Supplementary material for: YL143, a novel mutant selective irreversible EGFR inhibitor, overcomes EGFRL858R, T790M‐mutant resistance in vitro and in vivo
Source: Cancer Med. 2018 Mar 13;7(4):1430–9. doi: 10.1002/cam4.1392 (PMC5911580; doi:10.1002/cam4.1392)
Supplement: Supplementary file 2 — Table S1. Anti‐proliferative activities of YL143 and drugs in BaF3 stable cells. [file CAM4-7-1430-s002.docx]

**Table S1. Anti-proliferative activities of YL143 and drugs.**

| **μM (AV±SD)** | **BaF3** | **BaF3-EGFR ^L858R^** |
| --- | --- | --- |
| **Gefitinib** | >10 | 0.010±0.010 |
| **AZD9291** | 6.174±2.979 | 0.024±0.025 |
| **CO1686** | 2.341±1.665 | 0.069±0.048 |
| **YL143** | >10 | 0.071±0.041 |

The anti-proliferative activities of the compounds were evaluated using CCK-8 assay. The data were means from at least four independent experiments.
