# Supplementary material for: YL143, a novel mutant selective irreversible EGFR inhibitor, overcomes EGFRL858R, T790M‐mutant resistance in vitro and in vivo
Source: Cancer Med. 2018 Mar 13;7(4):1430–9. doi: 10.1002/cam4.1392 (PMC5911580; doi:10.1002/cam4.1392)
Supplement: Supplementary file 3 — Table S2. In vitro kinases activities of YL143. [file CAM4-7-1430-s003.docx]

**Table S2. In vitro kinases activities of YL143**

| **Kinase** (**μM**) | **AKT1** | **AKT2** | **AKT3** | **PDGFRA** | **PDGFRB** | **EGFR** |
| --- | --- | --- | --- | --- | --- | --- |
| IC_50_ | >10 | >10 | >10 | >10 | >10 | 3.531E-08 |
| **Kinase** (**μM**) | **ZAK** | **CSF1R** | **FLT3** | **TRKA** | **Abl** | **Kit** |
| IC_50_ | >10 | >10 | >10 | >10 | >10 | >10 |

Kinase activity assays were performed using the FRET-based Z′-Lyte assay according to the manufacturer’s instructions. The compounds were incubated with the kinase reaction mixture for 1.5 hrs before measurement. The data are means from two independent experiments and the variations are below 20%.
